# COMBINING ASSESSMENT OF MUSCLE STRENGTH WITH MYOPENIA BETTER PREDICTS EARLY POSTOPERATIVE COMPLICATIONS AFTER PANCREATICODUODENECTOMY

**DOI:** 10.1590/S0004-2803.24612024-136

**Published:** 2025-09-05

**Authors:** Saurabh MESHRAM, Santosh IRRINKI, Vishal SHARMA, Pankaj GUPTA, Vikas GUPTA, Thakur Deen YADAV, Rajesh GUPTA, Harjeet SINGH

**Affiliations:** ¹ Post Graduate Institute of Medical Education and Research, Department of General Surgery, Chandigarh, India.; ² Post Graduate Institute of Medical Education and Research, Department of Gastroenterology, Chandigarh, India.; ³ Post Graduate Institute of Medical Education and Research, Department of Radiodiagnosis, Chandigarh, India.; 4 Department of GI Surgery, HPB and Liver Transplantation, Post Graduate Institute of Medical Education and Research, Chandigarh, India.

**Keywords:** Sarcopenia, pancreaticoduodenectomy, postoperative pancreatic fistula, surgery, prehabilitation, Sarcopenia, duodenopancreatectomia, fístula pancreática pós-operatória, cirurgia, pré-habilitação

## Abstract

**Background:**

Pancreaticoduodenectomy (PD) is a complex procedure with significant postoperative morbidity. Associated sarcopenia could be a potential risk for increased post-operative complications.

**Methods:**

Patients who had undergone pancreaticoduodenectomy bet­ween July 2019 to December 2020 were included in the study. Preope­rative comprehensive sarcopenia assessment was done by hand grip strength test, Dual energy X-ray absorptiometry (DEXA) scan and gait speed test. Only myopenia was also assessed by DEXA scan in all the patients. Post-operative outcomes were recorded and the association of preoperative sarcopenia with postoperative complications were analyzed.

**Results:**

Of 47 patients assessed, 36 patients were finally included (Median age -58 years (IQR-51,68) years, 26 male). The five (13.8%) had sarcopenia confirmed on comprehensive assessment. Thirteen (36.5%) patients had myopenia on DEXA assessment. The major Clavien-Dindo complications were significantly higher in sarcopenia (40% vs 6.6%, *P*=0.04) and similarly, grade C DGE (40% vs 0, *P*=0.04) was also more frequent in patients with sarcopenia. The patients with myopenia only did not have a significant correlation with post-operative complications. (15.4% vs 8.7% *P*=0.66).

**Conclusion:**

Comprehensive assessment using muscle strength and muscle quantity is essential for sarcopenia diagnosis. Preoperative sarcopenia is a significant risk factor for post-operative complications.

## HIGHLIGHTS

• Pancreaticoduodenectomy (PD) is a complex surgical procedure with significant morbidity.

• Associated sarcopenia could be potential risk for increased post operative complications

• Comprehensive assessment of sarcopenia is an essential tool to predict post operative complication after PD.

## RESUMO

**Contexto:**

A duodenopancreatectomia (DP) é um procedimento complexo com morbidade pós-operatória significativa. A sarcopenia associada pode ser um risco potencial para aumento de complicações pós-operatórias.

**Métodos:**

Pacientes submetidos à duodenopancreatectomia entre julho de 2019 e dezembro de 2020 foram incluídos no estudo. A avaliação abrangente da sarcopenia pré-operatória foi feita por teste de força de preensão manual, absorciometria de raios-X de dupla energia (DEXA) e teste de velocidade da marcha. Apenas a miopenia também foi avaliada por DEXA em todos os pacientes. Os resultados pós-operatórios foram registrados e a associação de sarcopenia pré-operatória com complicações pós-operatórias foi analisada.

**Resultados:**

Dos 47 pacientes avaliados, trinta e seis pacientes foram finalmente incluídos (idade mediana -58 anos (IQR-51,68) anos,26 do sexo masculino). Os cinco (13,8%) tiveram sarcopenia confirmada na avaliação abrangente. Treze (36,5%) pacientes apresentaram miopenia na avaliação do DEXA. As principais complicações de Clavien-Dindo foram significativamente maiores na sarcopenia (40% vs 6,6%, *P*=0,04) e, da mesma forma, o DGE grau C (40% vs 0, *P*=0,04) também foi mais frequente em pacientes com sarcopenia. Os pacientes com miopenia apenas não apresentaram correlação significativa com complicações pós-operatórias. (15,4% vs 8,7% *P*=0,66).

**Conclusão:**

A avaliação abrangente usando força muscular e quantidade muscular é essencial para o diagnóstico de sarcopenia. A sarcopenia pré-operatória é um fator de risco significativo para complicações pós-operatórias.

## INTRODUCTION

Pancreaticoduodenectomy (PD) is a complex surgical procedure and is associated with significant post-operative morbidity. In the recent decade, with advancements in perioperative care and improved surgical techniques, a significant decrea­se in post-operative morbidity and mortality has been noted^1^ ^,^ ^2^. Despite this, the reported morbidity at high-volume centers is 40-55%^3^ ^,^ ^4^. Postoperative complications can delay adjuvant treatment, increase hospital stay, increase treatment costs, and result in poor quality of life^5^ ^,^ ^6^.

Many possible risk factors have been reported to result in post-operative complications following PD, including male gender, preoperative biliary drainage, obesity, intraoperative blood loss and soft pancreatic stump^7^ ^,^ ^8^. Early identification of these risk factors helps in risk stratification, better patient counselling and adequate time for prehabilitation for optimal outcomes. Sarcopenia has been reported as an important risk factor for occurrence post- operative complications following various surgeries^9^ ^,^ ^10^ ^,^ ^11^. Only a few studies have evaluated the role of sarcopenia in predicting complications after pancreaticoduodenectomy. The majority of these studies are limited by retrospective design and are focused only on quantitative muscle mass assessment using CT parameters rather than a complete assessment that considers muscle function^12^ ^,^ ^13^ ^,^ ^14^ ^,^ ^15^.

Hence, we planned a comprehensive assessment of sarcopenia combining a DEXA scan (for muscle mass), hand grip method (muscle function) and Gait speed test (physical performance). We aimed to prospectively see the effect of sarcopenia as defined per the revised European definition of sarcopenia on post-operative complications after pancreaticoduodenectomy^16^.

## METHODS

### Study design and population

This study is a single-centre prospective study conducted at a tertiary care centre of North India between July 2019 to December 2020. The study was approved by the institutional ethics committee (INT/IEC/2020/SPL-334). All the patients who had undergone pancreaticoduodenectomy (PD) for various indications during the study duration were included. All participants provided written informed consent. Patients with an ASA grade of more than three, who could not perform the required qualitative assessment intended in this study due to underlying issues like cerebrovascular stroke, arthritis, or physical disabilities and who were not fit to undergo PD, were excluded from the study. Clinical demographic features including comorbidities, and laboratory parameters were recorded. All patients underwent preoperative cross-sectional imaging (CECT/ MRI abdomen) for staging. Preoperative biliary drainage was done if indicated.

### Assessment of sarcopenia

#### Assessment of hand grip

Muscle strength assessment was performed using Hand grip strength (HGS) using a handheld dynamometer (Jamar hydraulic hand dynamometer). The patient was asked to sit comfortably in a standard chair with back support. The shoulder was adducted and the elbow was in 90-degree flexion. The wrist was in a neutral position with the thumb facing upward. The handlebar was adjusted as per patient comfort. The procedure was demonstrated once by the investigator. The patient was asked to press as tightly as possible for 3 to 5 seconds. The procedure was repeated 3 times with each arm and the maximum reading. The average of three readings from the dominant hand was reported as the final result. The rest period of 1 minute was given between the two consecutive readings. The cut-off value of sarcopenia was <27.5 in males and <18 kg in females as derived from a population-level study done in Chandigarh^17^.

### Assessment of muscle mass

Quantitative assessment of the muscle quantity was performed using a dual-energy X-ray absorptiometry (DEXA) scan using appendicular skeletal mass index (ASM). ASM has been adjusted for the body size using the height adjustment formula (ASM/ht2). DEXA has been performed using Hologic Discovery W and the measurements are analysed using standard software. Sarcopenia was diagnosed in the patients who had probable sarcopenia and in addition, had ASM/ht2 value of less than <6.11 kg/m^2^ in men and <4.61 kg/m^2^ in women^17^.

### Assessment of physical performance

The physical performance was assessed using a Gait speed test. The manual assessment of gait speed was performed during a 4 - meter walk done by the patient. A straight-marked course was used. The manual measurement was made by a stopwatch. Instructions to walk at the usual pace from a still-standing position behind the starting line were provided to participants. Timing started at the first foot movement and ended when a foot completely crossed the finish line. Gait speed was calculated by the formula 4/ (time taken to complete the walk-in seconds). Gait speed was expressed in meters/seconds. The Gait speed test was considered abnormal if it was <0.8 m/s^17^ ^,^ ^18^.

### Definition of sarcopenia

Sarcopenia was defined as per the “updated European Working Group on Sarcopenia in Older People” (EWGSOP2)^16^. Patients with reduced hand grip strength were defined as probable sarcopenia. Patients with combined reduced muscle strength and abnormal ASM/ht2 were considered as confirmed sarcopenia. The patients were considered severely sarcopenic if their gait speed was <0.8 m/s. Myopenia is defined as abnormal ASM/ht2 values on DEXA scan (<6.11 kg/m2 in men and <4.61 kg/m2 in women).

### Surgical procedure

All the surgical procedures were performed by experienced hepato- biliary - pancreatic surgeons. After the resection, pancreatic stump reconstruction was done with pancreaticojejunostomy (PJ). End to side hepaticojejunostomy was done 10-15 cm from PJ anastomosis. Gastrojejunostomy or duodenojejunostomy was done using a stapler or hand sewn as per surgeon’s discretion. Soft drains were placed adjacent to anastomosis. All patients were encouraged for early ambulation and enteral nutrition was started on post-operative day one.

### Definition of post-operative complications

Post-operative complications were graded as per Clavien- Dindo classification into minor (Grade I and II) and major complications (Grade III, IV& V)^19^.The postoperative pancreatic fistula, delayed gastric emptying, post-operative pancreatic haemorrhage was defined as per International Study Group for Pancreatic Surgery (ISGPS) definitions^20^ ^-^ ^22^. Surgical site infections are defined as per the Centre of disease control and Prevention (CDC) guidelines^23^. The post-operative complication occurrence was compared in patients with sarcopenia and no sarcopenia.

### Statistical analysis

Statistical product and service solutions (SPSS) version 23 was used for data analysis. The continuous data were expressed in Mean + Standard deviation (SD) or as median and interquartile range (IQR). The categorical variables were expressed as frequencies and percentages. For compassion continuous variables between the groups Mann - Whitney U test was used. Categorical variables were compared by chi-square test if the value of all cells in the expected table was >5 else Fisher’s exact test was applied. The *P*-value of <0.05 was considered as statistically significant.

## RESULTS

A total of 47 patients were assessed for enrolment during the study period. Eleven patients were excluded (two patients were found to have unresectable disease on preoperative imaging, one denied consent, six patients could not undergo DEXA scan and two had metastatic disease diagnosed intraoperatively). Hence, 36 patients were finally included in the study.

The demographic profile, intraoperative characteristics and post-operative outcomes were summarised in Table 1. The median age of the study population was 58 (IQR-51,68) years and 26 (72.2%) were male. The median BMI was 22.2 (IQR-19.1, 24.1) kg/m2. Eighteen patients required preoperative biliary drainage. The indication of pancreaticoduodenectomy was a periampullary tumor in 24 (66.6%), pancreatic head adenocarcinoma 8 (22.2%), pancreatic head adenocarcinoma with chronic pancreatitis 1 (2.7%), pancreatic head neuroendocrine tumor 2 (5.5%) and pancreatic head solid pseudopapillary neoplasm 1 (2.7%) patient. The median post operative stay was 11.5 (IQR-9.0, 16.0) days and major complications were noted in 11.1% (4/36). The clinically relevant postoperative pancreatic fistula (POPF), delayed gastric emptying (DGE) and post-pancreatectomy hemorrhage (PPH) were noted in 11.1% (4/36), 58.3% (21/36) and 2.8 (1/36) respectively.

**TABLE 1:** Demographics and operative characteristics of the study population.

Characteristic	Total study population (n=36)	Sarcopenia (n=5)	No sarcopenia (n=31)	***P* value**
Age, median (IQR) years	58 (51,68)	65 (52,73)	58 (51,66)	1.00
Sex (male), n (%)	26 (72)	5 (100)	21 (67.7)	0.13
BMI, median (IQR) kg/m^2^	22.2 (19.1,24.1)	19 (18.1,22)	23.2 (19.6,24.4)	0.36
Diabetes mellitus n (%)	8(22.2)	0	8(25.8)	0.19
Hypertension n (%)	9 (25)	1 (20)	8 (25.8)	0.78
Smoking n (%)	10 (27)	3 (60)	7 (22.6)	0.08
History of alcohol intake n (%)	9 (25)	3 (60)	6 (19.3)	0.05
Median preoperative serum albumin (gm/dL)	3.8 (3.4-4.1)	3.5 (3.04, 4)	3.9 (3.4,4.2)	1.00
Surgery duration, median (IQR) min	300 (240,360)	360 (240,420)	300 (200,400)	0.58
Blood loss, median (IQR) ml	275 (200-400)	200 (125, 400)	300 (240,360)	1.0
Pancreatic duct diameter, median (IQR) mm	4 (3,6)	4 (4,8)	4.5 (3,6)	0.73
Pancreatic stump consistency (%)				
Soft	9(25)	2(40)	7 (22.6)	0.56
Firm	27(75)	3(60)	24(77.4)	
Pancreatic pathology				
PDAC	9 (25)	2 (40)	7 (22,6)	0.56
Other	27 (75)	3 (60)	24 (77.4)	

### Assessment of sarcopenia

All the 36 patients were evaluated with the handgrip assessment and 11 (30.5%) patients were found to be below the predetermined cut-off level. The Median handgrip strengths were 27 (IQR-24,32) and 21 (IQR-20,22) kg among the males and females respectively. Five patients (13.8% of total) out of the 11 patients had sarcopenia confirmed on the DEXA scan. The median ASMI/ht2 was 6.23(IQR-5.73, 6.81) and 5.5(IQR-5.2, 6.27) kg/m2 among the males and females respectively. All the five patients who were confirmed to have sarcopenia on DEXA were found to have severe sarcopenia based on the Gait speed test (median - 0.7 m/sec).

### Association of sarcopenia and postoperative complications

The major Clavin-Dindo complications were more common in patients with sarcopenia (40% vs 6.5%, *P*=0.04) and similarly, grade C DGE (40% vs 0, *P*=0.04) was also more frequent in patients with sarcopenia. The occurrence of POPF (60% vs 45.2%, *P*=0.53) was also higher in sarcopenic group but it was not statistically significant. Table 2 summarises the analysis of various complications between the sarcopenia and no sarcopenia patients.

**TABLE 2:** Comparison of postoperative outcomes among the patients with and without sarcopenia.

Outcomes	Total population (n=36)	Sarcopenia Group (n=5)	No sarcopenia Group (n=31)	***P* value**
Post-operative Hospital stay, median (IQR) days	11.5 (9,16)	14 (9,17.5)	11(9,16)	1.0
Wound Infection, n (%)	12 (33.3)	3 (60)	9 (29)	0.17
Intra-abdominal collection, n (%)	4 (11.1)	2 (40)	2 (6.5)	0.02
POPF, n (%)	17 (47.2)	3 (60)	14 (45.2)	0.53
Clinically relevant POPF, n (%)	4 (11.1)	1 (20)	3 (9.7)	0.73
DGE n (%)	21 (58.3)	4 (80)	17 (54.8)	0.004
Grade A	9 (25)	1 (20)	8 (25.8)	
Grade B	10 (27)	1 (20)	9 (29)	
Grade C	2 (5.6)	2 (40)	0	
POPH n (%)	1 (2.8)	0	1 (3.2)	0.68
Clavien-Dindo major complications, n (%)				
Grade III	4 (11.1)	2 (40)	2 (6.5)	0.04
Grade IV	0	0	0	
Grade V	0	0	0	

### Comparison of sarcopenia between new definition and old definition

With the use of DEXA scan 13 out of 36 patients had ASM/ht2 values below the cut-off level. TABLE 3 shows the comparison of complications with the use of old (myopenia alone) and new definitions of sarcopenia. The new definition had a significant correlation of sarcopenia with the occurrence of complications.

## DISCUSSION

In the present study, 5 out of 36 patients had preoperative sarcopenia. Four patients who developed major complications were sarcopenic. The patients with sarcopenia had higher incidence of POPF and DGE. Similarly, the risk of major postoperative complications were also higher in patients with sarcopenia. The sarcopenia defined by only radiological parameters overestimates its incidence and is a poor predictor of complications.

**FIGURE 1.**
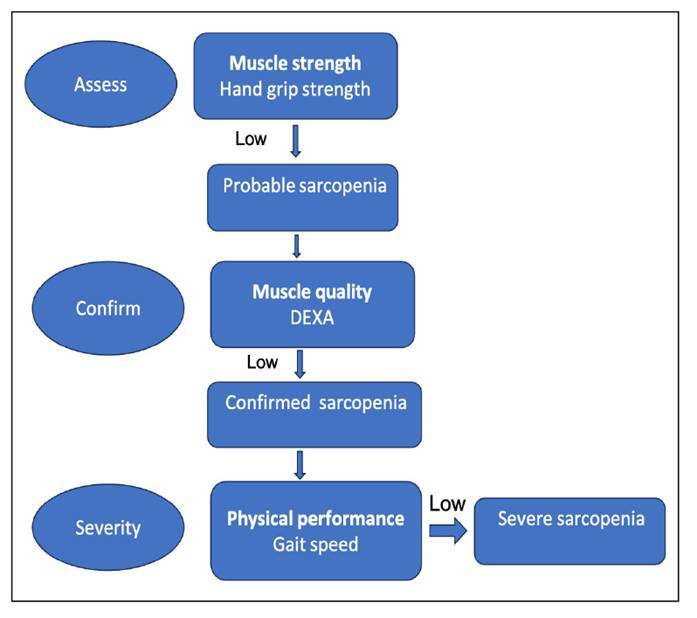
Algorithm for making a diagnosis and quantifying severity of sarcopenia.

For optimal surgical outcomes, the patient’s underlying physiological status is at most important. Sarcopenia is an objective measure to assess the underlying physiological status. Traditionally, in the majority of studies, the sarcopenia has been assessed only by cross-sectional imaging by estimating lean muscle mass^12^ ^-^ ^15^. The presence of both decreased skeletal muscle mass and low muscle function are essential for the definition of sarcopenia. In 2019 revised EWGSOP2 guidelines for sarcopenia were published which include muscle quantity, muscle strength and physical performance by means of radiological and physical tests^16^. In the present study, these factors were included in the assessment of sarcopenia (Figure 1). The pathophysiology of the preoperative sarcopenia and associated post-operative complications is not very clear. It has been hypothesised, that sarcopenia being a proinflammatory state results in poor immune response and impaired wound healing. There is associated dysregulation in hypertrophy and regeneration, which further lead to loss of muscle mass and muscle function^24^ ^,^ ^25^.

There is heterogeneity in the published literature regarding the role of sarcopenia in the occurrence of postoperative complications^12^ ^,^ ^13^ ^,^ ^26^. In a study by Peng P^13^, sarcopenia did not show any association with postoperative morbidity. Similarly, in a systematic review, only five of seventeen studies showed an association of sarcopenia with postoperative pancreatic fistula after pancreaticoduodenectomy^27^. On the other hand Amini N et al.^12^ showed a significant correlation of sarcopenia measured by total psoas volume (TPV) with post morbidity. Similarly, Joglekar et al.^14^ reported that sarcopenia was an independent prognostic factor of the surgical outcome by measuring psoas muscle density in patients of pancreatic cancer after PD. The possible explanation for this heterogeneity is, different sites of skeletal muscle measurement, different cuff-off values to define sarcopenia, heterogeneous study groups, retrospective data and use of only radiological tools to diagnose sarcopenia, and not using muscle strength as a part of the definition of sarcopenia. In this study also, the incidence of radiological sarcopenia (measured by DEXA) was 36.5% and showed poor association with complications using this definition (Table 3).

**TABLE 3:** Comparison between new and old definition of sarcopenia.

	Old definition (myopenia diagnosed only with DEXA scan)	New definition
Probable sarcopenia, n (%)	NA	11(30.5)
Sarcopenia, n (%)	13 (36.5)	5 (13.8)
Severe sarcopenia, n (%)	NA	5 (13.8)
Difference in major complication between no sarcopenia and sarcopenia groups	Sarcopenia - 15.4 %	Sarcopenia - 40%
	No sarcopenia - 8.7%	No sarcopenia - 6.5%
	*P*=0.66	*P*=0.04

In the current study, we found an increased incidence of major complications, CRPOPF and DGE in patients with sarcopenia. In our study, 11 out of 36 patients showed reduced hand grip strength and finally, five patients had confirmed sarcopenia after DEXA scan. The hand grip strength test can be used as an initial screen for further evaluation of sarcopenia. It is non-invasive and very easy to perform bed­side. Cruz-Jentoft et al. 16 also advocated the same in their algorithmic approach to diagnose probable cases of sarcopenia. In our study, we used DEXA for quantification of muscle mass. DEXA has been reported most effective and reproducible method of estimation of lean mass^16^. In the majority of the studies CT scan is the most commonly used tool for the estimation of muscle mass. Possibly because of easy availability and the same can be used for staging primary disease.

In our centre, we carefully evaluate and optimise patients for nutritional status before pancreaticoduodenectomy. We delay surgery, if possible if the serum albumin level is less than <3gm%. We do nutritional counselling and try to ensure the nutritional demands are met. However, before contemplating the present study sarcopenic assessment was not routinely performed at our centre. We believe detection of sarcopenia should be an essential component of preoperative workup. If sarcopenia is diagnosed, patients should be planned for nutritional support, and preoperative rehabilitation including muscle strengthening and improving physical performance.

Our study has a few limitation limitations including a small sample size and single-centre study. Being a single centre, the patient population was all Indian, there is concern about the reproducibility of the same results in other ethnic groups or populations. Despite these limitations, a major strength of our study is being prospective data and assessment of sarcopenia by muscle mass and muscle function in which cuff value of diagnosis was taken from Indian population.

In conclusion, deficiency in muscle strength along with muscle quantity defines true sarcopenia. The postoperative complication after pancreaticoduodenectomy was significantly higher with sarcopenia as defined by EWGSOP2. So Early diagnosis of sarcopenia is crucial and preoperative intervention in the form of Prehabilitation in this high-risk group may help in decreasing postoperative complications.

## References

[1] Cameron JL, Pitt HA, Yeo CJ, Lillemoe KD, Kaufman HS, Coleman J (1993). One hundred and forty-five consecutive pancreaticoduodenectomies without mortality. Ann. Surg.

[2] Singh H, Krishnamurthy G, Kumar H, Gorsi U, Kumar-M P, Mandavdhare H (2020). Effect of bile duct clamping versus no clamping on surgical site infections in patients undergoing pancreaticoduodenectomy: a randomized controlled study. ANZ J Surg.

[3] Addeo P, Delpero JR, Paye F, Oussoultzoglou E, Fuchshuber PR, Sauvanet A (2014). French Surgical Association (AFC). Pancreatic fistula after a pancreaticoduodenectomy for ductal adenocarcinoma and its association with morbidity: amulticentre study of the French Surgical Association. HBP.

[4] Mohan A, Gupta R, Yadav TD, Gupta V, Sharma V, Mandavdhare H (2022). Association of Intra-Operative Bile Culture with Post-Operative Complications after Pancreaticoduodenectomy. Surg Infect.

[5] Wu W, He J, Cameron JL, Makary M, Soares K, Ahuja N (2014). The impact of postoperative complications on the administration of adjuvant therapy following pancreaticoduodenectomy for adenocarcinoma. Ann Surg Oncol.

[6] Kanwat S, Singh H, Sharma AK, Sharma V, Gupta P, Gupta V (2023). Pancreatic Dysfunction and Reduction in Quality of Life Is Common After Pancreaticoduodenectomy. Dig Dis Sci.

[7] House MG, Fong Y, Arnaoutakis DJ, Sharma R, Winston CB, Protic M (2008). Preoperative predictors for complications after pancreaticoduodenectomy: impact of BMI and body fat distribution. J Gastrointest Surg.

[8] Sushma N, Gupta P, Kumar H, Sharma V, Mandavdhare H, Kumar-M P (2020). Role of ultrasound shear wave elastography in preoperative prediction of pancreatic fistula after pancreaticoduodenectomy. Pancreatology.

[9] Voron T, Tselikas L, Pietrasz D, Pigneur F, Laurent A, Compagnon P (2015). Sarcopenia Impacts on Short and Long-term Results of Hepatectomy for Hepatocellular Carcinoma. Ann Surg.

[10] Meza-Junco J, Montano-Loza AJ, Baracos VE, Prado CMM, Bain VG, Beaumont C (2013). Sarcopenia as a Prognostic Index of Nutritional Status in Concurrent Cirrhosis and Hepatocellular Carcinoma. J Clin Gastroenterol.

[11] Otsuji H, Yokoyama Y, Ebata T, Igami T, Sugawara G, Mizuno T (2015). Preoperative sarcopenia negatively impacts postoperative outcomes following major hepatectomy with extrahepatic bile duct resection. World J Surg.

[12] Amini N, Spolverato G, Gupta R, Margonis GA, Kim Y, Wagner D (2015). Impact Total Psoas Volume on Short- and Long-Term Outcomes in Patients Undergoing Curative Resection for Pancreatic Adenocarcinoma: a New Tool to Assess Sarcopenia. J Gastrointest Surg.

[13] Peng P, Hyder O, Firoozmand A, Kneuertz P, Schulick RD, Huang D (2012). Impact of sarcopenia on outcomes following resection of pancreatic adenocarcinoma. J Gastrointest Surg.

[14] Joglekar S, Asghar A, Mott SL, Johnson BE, Button AM, Clark E (2015). Sarcopenia is an independent predictor of complications following pancreatectomy for adenocarcinoma. J Surg Oncol.

[15] Nishida Y, Kato Y, Kudo M, Aizawa H, Okubo S, Takahashi D (2016). Preoperative sarcopenia strongly influences the risk of postoperative pancreatic fistula formation after pancreaticoduodenectomy. J Gastrointest Surg.

[16] Cruz-Jentoft AJ, Bahat G, Bauer J, Boirie Y, Bruy ere O, Cederholm T (2019). Sarcopenia: revised European consensus on definition and diagnosis. Age Aging.

[17] Pal R, Aggarwal A, Singh T, Sharma S, Khandelwal N, Garg A (2020). Diagnostic cut-offs, prevalence, and biochemical predictors of sarcopenia in healthy Indian adults: The Sarcopenia-Chandigarh Urban Bone Epidemiological Study (Sarco-CUBES). Eur Geriatr Med.

[18] Maggio M, Ceda GP, Ticinesi A, De Vita F, Gelmini G, Costantino C (2016). Instrumental and Non-Instrumental Evaluation of 4-Meter Walking Speed in Older Individuals. PLoS ONE.

[19] Dindo D, Demartines N, Clavien P-A (2004). Classification of surgical complications: a new proposal with evaluation in a cohort of 6336 patients and results of a survey. Ann Surg.

[20] Bassi C, Marchegiani G, Dervenis C, Sarr M, Abu Hilal M, Adham M (2017). The 2016 update of the International Study Group (ISGPS) definition and grading of postoperative pancreatic fistula: 11 Years After. Surgery.

[21] Wente MN, Bassi C, Dervenis C, Fingerhut A, Gouma DJ, Izbicki JR (2007). Delayed gastric emptying (DGE) after pancreatic surgery: A suggested definition by the International Study Group of Pancreatic Surgery (ISGPS). Surgery.

[22] Wente MN, Veit JA, Bassi C (2007). Postpancreatectomy hemorrhage (PPH) - an international study group of pancreatic surgery (ISGPS) definition. Surgery.

[23] Berríos-Torres SI, Umscheid CA, Bratzler DW, Leas B, Stone EC, Kelz RR (2017). Centers for Disease Control and Prevention Guideline for the Prevention of Surgical Site Infection, 2017. JAMA Surg.

[24] Cruz-Jentoft AJ, Sayer AA (2019). Sarcopenia. Lancet.

[25] Lutz CT, Quinn LS (2012). Sarcopenia, obesity, and natural killer cell immune senescence in aging: altered cytokine levels as a common mechanism. Aging.

[26] Aoki Y, Furukawa K, Suzuki D, Takayashiki T, Kuboki S, Takano S (2022). Influence of sarcopenia as defined by EWGSOP2 on complications after pancreaticoduodenectomy and on the prognosis of pancreatic head cancer: A prospective cohort study. Nutrition.

[27] Perra T, Sotgiu G, Porcu A (2022). Sarcopenia and Risk of Pancreatic Fistula after Pancreatic Surgery: A Systematic Review. J Clin Med.

